# Clinical and Pathological Features of Cutaneous Burns Caused by Mobile Phone Charging Devices

**DOI:** 10.7759/cureus.104030

**Published:** 2026-02-21

**Authors:** Sooyie Choi, Yeon Woo Jung, Eunsung Cho, Byung-Ho Oh

**Affiliations:** 1 Department of Dermatology, Galleria Dermatology Clinic, Gangdong-Songpa Branch, Seoul, KOR; 2 Department of Dermatology and Cutaneous Biology, Yonsei University College of Medicine, Seoul, KOR

**Keywords:** burn, charger, debridement, mobile phone, skin biopsy

## Abstract

We report two rare cases of cutaneous burns associated with portable charging devices: one from a micro-USB cable and the other from a power bank. Notably, even without exposed electrical terminals, skin injury can occur with brief contact with a charging phone. Portable power banks and other electronic devices may generate heat during prolonged use. These cases underscore the need for safety awareness regarding charging devices and caution against careless use while connected to a power supply, particularly during sleep or in situations with limited ability to respond to hazards.

## Introduction

Mobile phones and their charging devices have become indispensable in modern life, with billions of users worldwide relying on them for daily communication, work, and entertainment [1]. Despite their widespread use, the risk of cutaneous burns related to improper use of charging devices remains underrecognized. Previous reports have described thermal injuries resulting from prolonged contact with electrical devices, electrical leakage, or faulty batteries [2-6]. From a mechanistic perspective, cutaneous burns associated with electronic charging devices can be classified as thermal, electrical, or, less commonly, chemical burns. Thermal burns typically result from prolonged contact with overheated device components due to resistive heating, which may be exacerbated by continuous current flow during charging, inadequate heat dissipation, or battery-related overheating, particularly in portable power banks [2,5,6]. Electrical burns may occur in the setting of current leakage or insulation failure [4]. In rare cases, chemical burns have been reported, particularly with chargers that have exposed electrodes, where contact with sweat can induce electrochemical reactions leading to extreme pH changes and subsequent skin injury [3]. Here, we present two rare cases that illustrate the risk of burn injuries caused by portable electronic charging devices: one caused by a micro-USB charging cable and the other by a portable power bank.

## Case presentation

Case 1

**Figure 1:**
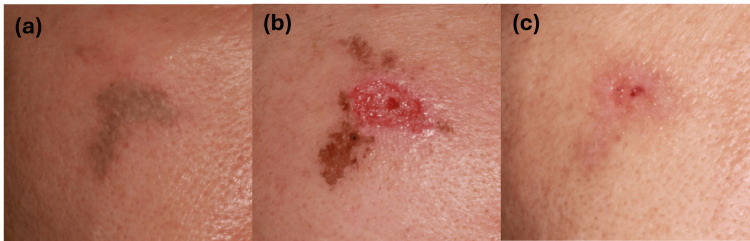
Clinical course of the burn lesion (Case 1) (a) Initial clinical presentation showing a linear grayish patch with peripheral erythema on the right cheek. (b) Immediate post-debridement appearance following surgical removal of necrotic tissue. (c) Clinical appearance at two-week follow-up after surgical debridement, showing nearly complete epithelization.

**Figure 2:**
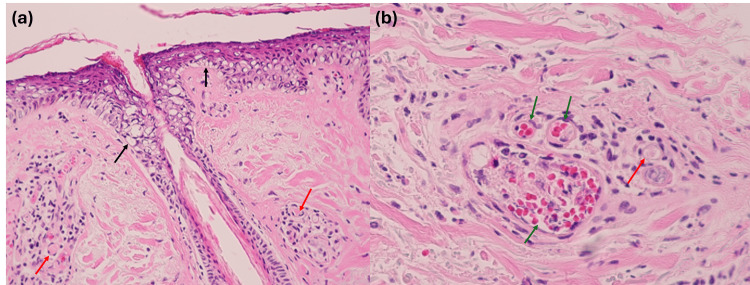
Histopathologic findings of the burn lesion (Case 1) (a) Epidermal thinning with hydropic changes extending into the infundibulum and isthmus (black arrows) and fibrin thrombi within blood vessels (red arrows) (H&E, ×100). (b) Perivascular inflammatory cell infiltration with erythrocyte aggregation (green arrows) and fibrin thrombi (red arrow), accompanied by thickened and eosinophilic collagen bundles (H&E, ×400). H&E: Hematoxylin and Eosin

A 55-year-old man presented with a grayish patch accompanied by a burning sensation on the right cheek one day after exposure (Figure 1). The injury occurred when his mobile phone, which was connected to a micro-USB charger, accidentally fell onto his face. Histopathological examination demonstrated epidermal thinning with hydropic changes extending to the infundibulum and isthmus (Figure 2). Dermal collagen bundle thickening with perivascular inflammatory cell infiltration, red blood cell aggregation, and fibrin thrombi were noted (Figure 2). The patient underwent debridement of necrotic tissue and achieved full recovery within two weeks (Figure 1). Based on the clinical circumstances involving contact with a charging device and the associated histopathological findings, the lesion was considered a thermal contact burn rather than an electrical or chemical injury.

Case 2

**Figure 3:**
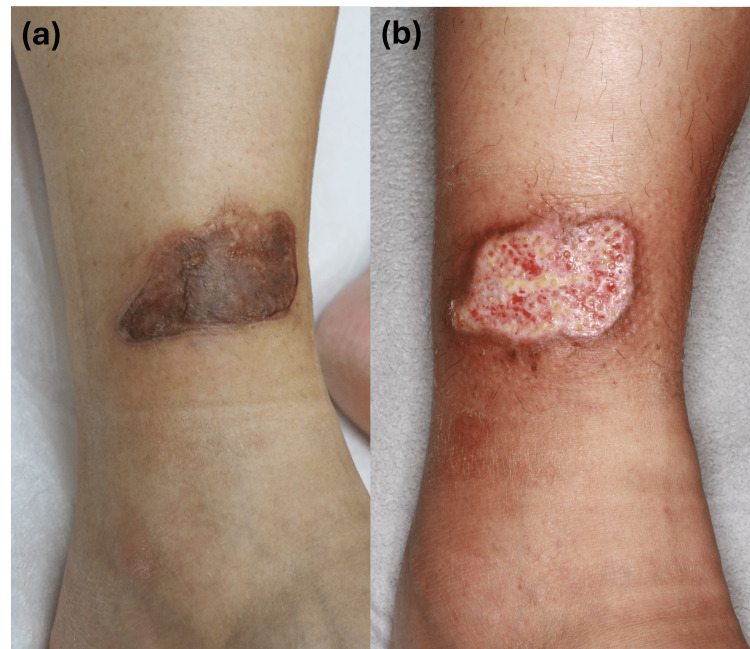
Clinical course of the burn lesion (Case 2) (a) Initial clinical presentation showing a grayish patch with bullae and an erythematous rim on the left shin. (b) Clinical appearance two weeks after surgical debridement, showing marked improvement during ongoing treatment with oral antibiotics.

**Figure 4:**
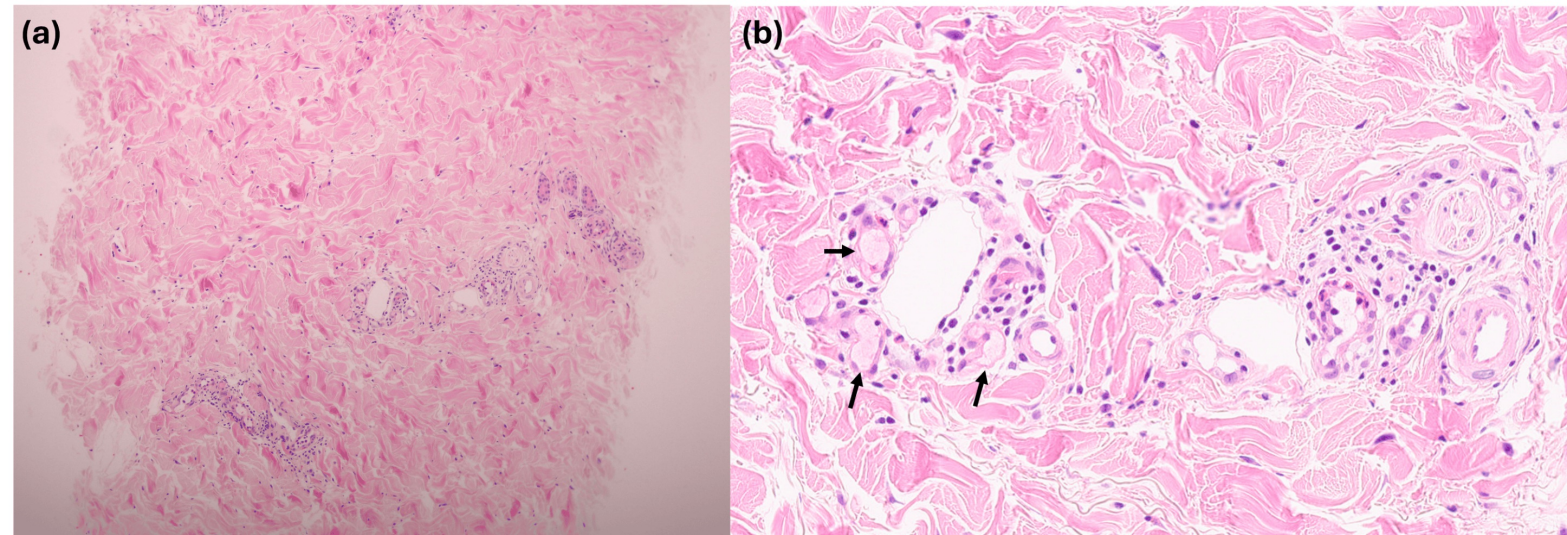
Histopathologic findings of the burn lesion (Case 2) (a) Perivascular and periadnexal inflammatory cell infiltration in the upper-to-deep dermis (H&E, ×100). (b) Fibrin thrombi within dermal vessels (black arrows) and perivascular cell infiltration (H&E, ×400). H&E: Hematoxylin and Eosin

A 38-year-old man presented with a palm-sized, grayish patch accompanied by bullae on the left ankle (Figure 3). Biopsy revealed perivascular and periadnexal inflammatory infiltrates in the upper-to-mid dermis with eosinophilic fibrinoid material within the vascular lumen (Figure 4). At the initial visit, he denied exposure to thermal sources; however, upon further questioning, he recalled that he had slept with a portable power bank beneath his leg while it was charging. Surgical debridement was performed approximately one month after the initial presentation, followed by oral antibiotic therapy. The lesion showed gradual improvement over a two-month period (Figure 3). Given the prolonged skin contact during charging and the absence of electrical shock or battery explosion, the injury was interpreted as a thermal contact burn.

## Discussion

Although skin biopsy is not routinely performed in clinical practice for cutaneous burns, several histopathologic findings may raise suspicion for burn-related injury and help assess the depth of necrosis and severity of injury [7,8]. Early epidermal and adnexal changes include coagulative necrosis with nuclear pyknosis, perinuclear halo formation, and karyorrhexis. Dermal injury is characterized by refractile eosinophilia and fusion of necrotic collagen fibers, while vascular alterations include erythrocyte aggregation, fibrin thrombus formation, and endothelial necrosis, reflecting injury to the microvasculature. Previous studies have identified stasis of dermal blood flow as a characteristic feature of thermal injury and have experimentally demonstrated that low-temperature, prolonged thermal exposure can induce vasculopathic changes [9]. Accordingly, the prominent vasculopathic findings observed in our two cases, including fibrin thrombus formation, may be regarded as supportive evidence for a diagnosis of thermal burn injury.

From a mechanistic standpoint, cutaneous burns associated with electronic devices may arise from thermal, electrical, or chemical mechanisms, which can partially overlap. In our cases, several factors argue against a primary electrical or chemical etiology. Electrical burns typically involve epidermal electrical breakdown and vaporization following direct current transmission, often resulting in sharply demarcated entry wounds, none of which were observed clinically or histologically [10]. Likewise, chemical burns are characterized by prolonged exposure and ongoing tissue destruction that persists as long as residual chemical agents remain in the tissue, a clinical course not observed in our patients [11]. Collectively, these considerations support a predominantly thermal mechanism related to sustained low-grade heat exposure.

The first case involved a micro-USB charging cable (Samsung Electronics Co., Ltd., Suwon, Korea), a device not typically implicated in burns. Previous reports have mainly described burn injuries caused by Apple’s 5W Lightning cables (Apple Inc., Cupertino, CA, USA), in which exposed metallic prongs at the charging tip predispose users to chemical or electrical injuries [3,4]. Our case demonstrates that skin burns can also occur with a micro-USB charger, which lacks exposed prongs, underscoring that thermal injury may arise from transient contact with a charging device regardless of charger design.

The second case underscores that portable power banks, although widely marketed for convenience, can also pose risks. The surface temperature of such devices may increase with prolonged use, and sustained skin contact with this moderate heat can result in cutaneous burns [5]. Previous case reports and international regulatory recalls have documented similar injuries related to overheating or malfunctioning of such devices [6,12-14]. The U.S. Consumer Product Safety Commission (CPSC), for example, has repeatedly issued recalls of portable charging devices due to fire and burn hazards [12-14]. These reports align with our findings, suggesting that the danger is not confined to specific brands or charger types but is inherent to the widespread use of portable charging accessories.

From a clinical perspective, these cases highlight the importance of considering electronic device-related burns in the differential diagnosis of atypical cutaneous injuries. Unlike classic flame or scald burns, such injuries may present with subtle clinical clues, and careful occupational and lifestyle history-taking is essential. Integrating clinical history with histopathologic assessment can be valuable in distinguishing these cases from mimickers such as contact dermatitis or vasculitic processes. However, given the retrospective nature of these cases, detailed device specifications, such as the exact model, power output, charging mode, and precise duration of skin contact, could not be fully ascertained, which limits more granular mechanistic inference.

## Conclusions

These cases emphasize that charging devices, including commonly used micro-USB cables and portable power banks, can cause cutaneous burns through mechanisms of overheating or prolonged skin contact, even in the absence of exposed electrical terminals. Although rare, our report highlights an underrecognized cause of burn injury, and histopathologic confirmation proved useful in establishing the diagnosis. While the relative contributions of charger design, device malfunction, improper use, and prolonged skin contact cannot be definitively established from a limited number of cases, clinicians should be aware of these uncommon but potentially serious injuries, and safety education should be reinforced for the general public. Users should avoid using charging devices in bed, leaving them under bedding, or maintaining prolonged direct skin contact while connected to a power supply.

On a broader scale, our report underscores the need for continued consumer safety monitoring and proactive regulatory measures, as evidenced by international recall data. Given the ubiquity of mobile devices worldwide, increased awareness among clinicians, manufacturers, and users is essential to prevent future injuries associated with everyday electronic accessories.
